# Cancer Diagnosis of Microscopic Biopsy Images Using a Social Spider Optimisation-Tuned Neural Network

**DOI:** 10.3390/diagnostics12010011

**Published:** 2021-12-22

**Authors:** Prasanalakshmi Balaji, Kumarappan Chidambaram

**Affiliations:** 1 Department of Computer Science, Center for Artificial Intelligence, King Khalid University, Abha 62529, Saudi Arabia; 2 Department of Pharmacology, School of Pharmacy, King Khalid University, Abha 62529, Saudi Arabia; kumarappan@kku.edu.sa

**Keywords:** biopsy, cancer diagnosis, predictive models, neural network, optimisation

## Abstract

One of the most dangerous diseases that threaten people is cancer. If diagnosed in earlier stages, cancer, with its life-threatening consequences, has the possibility of eradication. In addition, accuracy in prediction plays a significant role. Hence, developing a reliable model that contributes much towards the medical community in the early diagnosis of biopsy images with perfect accuracy comes to the forefront. This article aims to develop better predictive models using multivariate data and high-resolution diagnostic tools in clinical cancer research. This paper proposes the social spider optimisation (SSO) algorithm-tuned neural network to classify microscopic biopsy images of cancer. The significance of the proposed model relies on the effective tuning of the weights of the neural network classifier by the SSO algorithm. The performance of the proposed strategy is analysed with performance metrics such as accuracy, sensitivity, specificity, and MCC measures, and the attained results are 95.9181%, 94.2515%, 97.125%, and 97.68%, respectively, which shows the effectiveness of the proposed method for cancer disease diagnosis.

## 1. Introduction

The burden of cancer was estimated to be 1.7 million new cases and 0.65 million deaths in 2019 in the United States [1]. As the mortality rate and incidence rate of cancer rise considerably, extending the survival rate is of great concern with the advancements in recent technologies [2]. The treatment of cancer in the present era involves various choices, and since 2016, the effectiveness of treatment methods has been enhanced considerably [3,4]. Despite the introduction of new strategies for diagnosing cancer, the uncertainties in the precision of diagnostics are unavoidable. Hence, patient-specific, favourable treatments must be developed for the accurate diagnosis of the disease. In addition, the enhancements in the accuracy of prediction assist doctors in planning treatments for patients, both on mental and physical levels. The primary clinical observations are advised to be connected with the use of traditional approaches based on the size of the tumour, the spread of cancer to other parts of the body, and nearby lymph nodes [5]. The enhancement in the accuracy of diagnosis using the conventional methods of AI technology remains a severe challenge for clinical researchers. The advancements in computer software have facilitated health scientists to collaborate closely over improvements in prognosis. The accuracy of such models is found to be higher as compared to empirical predictions. With the execution of artificial intelligence (AI), researchers have turned to developing models using AI algorithms to predict and diagnose cancer. These strategies presently play a crucial role in enhancing the accuracy of cancer recurrence, susceptibility, and survival predictions. The digital pathology area has developed noticeably over recent years, due to the technological improvements in machine learning algorithms and image processing and increases in computation power. As part of this field, many methods have been introduced to automatically analyse and classify medical images [6].

This paper introduces an AI model to diagnose cancer using microscopic biopsy images. In the first step, the input image is pre-processed through the Region of Interest (ROI) segmentation method. The image, in RGB form, is converted into a binary image to make it subject to watershed segmentation, where the nucleus-based morphological features are extracted. Then, the features, such as the area, perimeter, diameter, and compactness from the nucleus-based morphological features are extracted. The extracted features develop as a feature vector that acts as the input to the proposed neural network (NN) classifier, the weights of which are optimally tuned using the SSO algorithm. This assists in enhancing the performance of the NN classifier in diagnosing the input image as benign and malignant.

The contribution and the key topics discussed in this study are:

- The proposed design for an automated model to diagnose the microscopic biopsy images as benign or malignant.
- The optimal tuning of the weights of the NN classifier by the SSO algorithm plays a vital role in enhancing the performance of the proposed classification model.
- The proposed model is analysed with conventional methods in terms of performance measures such as accuracy, sensitivity, specificity, and Matthew’s correlation coefficient (MCC) to validate the effectiveness of the proposed strategy.

The rest of the paper is organised as follows. Section 2 presents the survey of the current strategies for cancer diagnosis, including the challenges associated with them. Section 3 describes the proposed NN model in the diagnosis of cancer. Section 4 deliberates the outcomes of the proposed model, and Section 5 concludes the paper.

## 2. Literature Survey

This section focuses on the existing methods of cancer diagnosis and the challenges associated with the current models that act as the motivation for developing the proposed model.

### 2.1. Related Works

The literature review of the existing methods of cancer diagnosis is stated. Irfan Ullah Khan et al. [7] developed a model for the early analysis of cervical cancer with the aid of a reduced risk set of features and three ensemble classification strategies, such as Random Forest (RF), extreme Gradient Boosting (XGBoost), and AdaBoost, along with an FF algorithm for a hyperparameter tuning process. The model achieved a poor sensitivity measure, which is considered the method’s major drawback. Subrata Bhattacharjee et al. [6] examined the stained microscopic biopsy images to carry out image treatment and extract the important features to be used as input to the support vector machine (SVM) model for the diagnosis of cancer diseases. However, the model cannot make exact predictions, as errors in one class affect diagnosis accuracy. Ghulam Murtaza et al. [3] created a consistent and more precise model that used minimum resources with the aid of a convolution neural network (CNN) model. However, it is a tedious task to gather images of all types of cancers with appropriate labels. Salah Alheejawi et al. [8] developed a technique for the regular measurement of PI values in i-67 stained biopsy images using a deep-learning algorithm. This model segments the nuclei and evaluates the PI by using a trained CNN model. The method can robustly segment the nuclei with reduced computational complexity and possesses an average error rate of less than 0.7% but does not work well on nuclei clusters. Some of the existing works that implement swarm algorithms for optimising the hyperparameters of networks include artificial bee colony (ABC) [9], ant colony optimisation (ACO) [10], the firefly algorithm (FA) [11,12], cuckoo search (CS) [13], the bat algorithm (BA) [14], the whale optimisation algorithm (WOA) [15], elephant herding optimisation (EHO) [16,17,18], and many others [19,20,21,22]. Several swarm intelligence and evolutionary algorithms have been applied to the ANN hyperparameters’ optimisation to develop an automatic framework that will generate an optimal or near-optimal ANN structure for solving a specific problem.

### 2.2. Challenges

The challenges associated with the existing methods of cancer diagnosis include:

- The DNN-based strategies, particularly the CNN-based methods, have solved the handcrafted extraction of features. However, when this model is trained from scratch, it needs more annotated images and requires very high resources [3].
- In breast biopsy, the breast cancer samples are taken and preserved into microscopic slides for manual evaluation. An expert pathologist carries out the microscopic analysis, and the conclusion is made after the agreement of more than two pathologists for enhanced diagnosis. However, it may need increased time for diagnosis, and there may be a disagreement of opinion among two pathologists [3].

## 3. Proposed Method of Cancer Diagnosis

**Figure 1:**
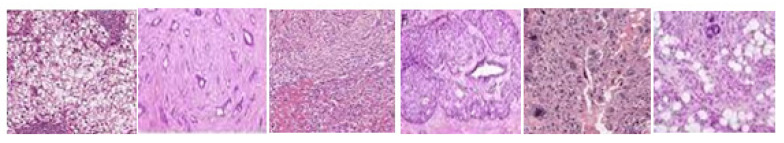
Sample Biopsy images.

An optimisation-tuned NN classifier is proposed in this research to classify cancer as benign or malignant. In the initial step, the microscopic biopsy images from the patients are subjected to pre-processing for the removal of artefacts present in the raw image data. To isolate the required object from the input image data, watershed segmentation of the image is performed after the conversion of the RGB image into a binary image. The object is then analysed using the proposed NN model with the provision of the features of the image as the training samples. The features, such as area, perimeter, diameter, and compactness, are extracted from the image, and from these features, the feature vector is developed through feature concatenation. The trained NN model, using the feature vector designed with the image features, is tested with the test data in such a way as to classify the cancer disease. Sample biopsy images are found in Figure 1.

### 3.1. Image Pre-Processing

The pre-processing step is the initial step of the proposed cancer disease diagnosis model, with the objective of removing the artefacts present in the images taken from the microscopic biopsy. The pre-processing step is necessary in such a way as to enhance the prediction accuracy of the proposed NN classifier. The raw microscopic biopsy images are normalised using the pre-processing step through ROI segmentation with the extraction of stroma, nuclei, and lumen in such a way to subject it to the successive steps of the diagnosis process [23]. The process of defining the ROI in an input frame is termed ROI segmentation. In texture and colour-based automatic ROI Segmentation, a specific region in the frame is selected and provided with its dimensions in the rectangle method to draw the rectangle-shaped ROI on the frame, classifying the image pixels as stroma, nuclei, and lumen.

**Figure 2:**
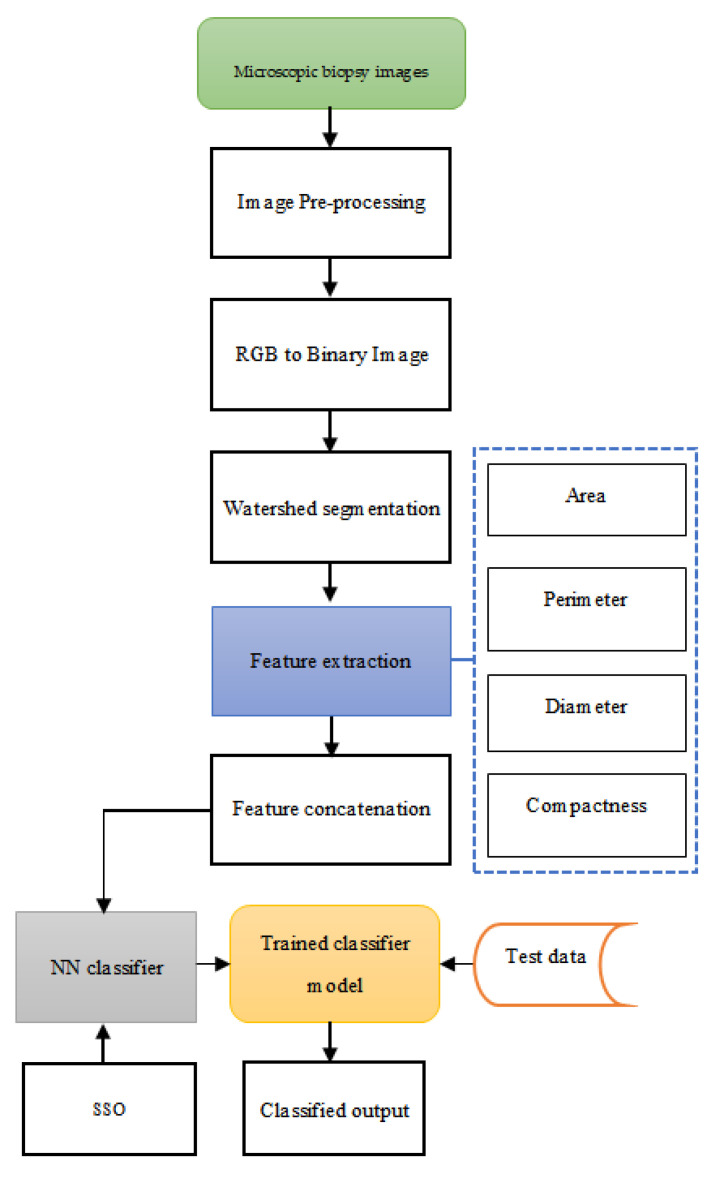
Schematic diagram of the proposed cancer disease diagnosis model.

The schematic representation of the proposed model is depicted in Figure 2.

### 3.2. Watershed Segmentation

The image, in RGB form, is converted into binary form in such a way as to describe the colour of the image with more recognisable comparisons, such as brightness, colour, and vitality [23]. The watershed segmentation is applied to the binary image to execute the process of object segmentation. The watershed segmentation process assists in the extraction of nucleus-based morphological features, from which the significant features are extracted. The watershed segmentation process aids in extracting nucleus-based morphological features that create a mask, from which the significant features are extracted. Segmenting the cells in the manner described above improves the accuracy of the cell count and the accuracy of the cell nucleus area statistics. To make statistics regarding the cell nucleus area even more accurate, any cell nuclei that are touching the border are removed, since most of those cells are more likely to be incomplete cells.

### 3.3. Feature Extraction and Concatenation

The significant features, such as area $a_{f}$, perimeter $p_{f}$, diameter $d_{f}$, and compactness $c_{f}$, are extracted from the nucleus based on the morphological characteristics of the input microscopic biopsy images. The extracted features are then concatenated to form the feature vector that acts as the input to the proposed NN classifier for a cancer diagnosis. The feature vector thus generated is represented as:

(1) $$F=\left\{a_{f},p_{f},d_{f}c_{f}\right\}$$

Thus, the concatenated feature acts as the input to the proposed SSO-based NN classifier in such a way as to execute the cancer diagnosis process.

### 3.4. Proposed Social Spider Optimisation Tuned Neural Network Classifier in Cancer Diagnosis

**Figure 3:**
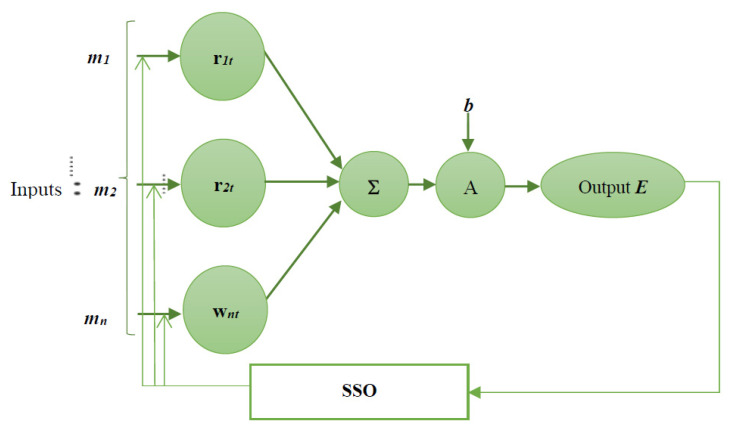
Structure of the proposed SSO-ANN.

An NN is a computational representation for performing tasks such as diagnosis, classification, decision making, etc. It consists of artificial neurons that act as a copy of human brain neurons. Neurons in the brain pass along signals to perform actions. Similarly, artificial neurons connect in a neural network to perform tasks. The connection between these artificial neurons is called weight. In other words, NN is a mathematical model designed based on biological neural networks with an interconnected gathering of artificial neurons, which processes data by a connection strategy for computation. NNs have arisen as an area of unusual opportunity for analysis, advancement, and application to different types of real-world problems in the past few years. NNs are the tremendously equivalent computing systems comprising a number of interconnections and simple processors. The principle of operation executed by humans is the basic tactic for developing NNs, as is the purpose of neurons in humans. NNs possess neuron layers in such a way to process the data which act as input. NNs consist of input multiplied by the weights to represent the flow of data. The mathematical functions involve evaluating the weights which in turn output the neuron’s activation function. The output of the NN can be adjusted as expected with the optimal tuning of weights, using the proposed SSO algorithm. The architecture of the NN is depicted in Figure 3.

The ANN architecture taken up here consists of conventional layers, as described below:

- The ANN is initialised with a sequential layer
  ann = tf.keras.models.Sequential()
- The fully connected input and the first hidden layer is added as a dense layer to the sequential layer with a uniform initialisation layer with a ‘relu’ activation function.
  ann.add(tf.keras.layers.Dense(units = 6, activation = ‘relu’))
- The second fully connected layer is added to the existing dense layer with the same ‘relu’ activation function.
  ann.add(tf.keras.layers.Dense(units = 6, activation = ‘relu’))
- Finally a fully connected output layer is added to the existing dense layer with the ‘sigmoid’ activation function.
  ann.add(tf.keras.layers.Dense(units = 1, activation = ‘sigmoid’))

The output of the ANN can be mathematically expressed as:

(2) $$E=A\left(\sum_{g=1}^{n}r_{g}m_{g}\right)+b$$

where A represents the activation function, $m_{g}$ is the input, and $r_{g}$ indicates the weight values. This output obtained from the ANN is fed to the SSO module for further optimisation.

The resulting ANN model is trained using the ‘Adam’ optimiser and a binary_crossentropy’ loss evaluation, meaning the ‘accuracy’ metric. The evaluation of the position of all possible positions in the proposed system is performed using the NN classifier. The significance of using an NN [24] is that the classifier evaluates in an automatic manner without requiring a human to check the process. The evaluation accuracy of the method relies on the proper usage of the proposed NN with noteworthy weight values using the proposed SSO algorithm.

### 3.5. Social Spider Optimisation Algorithm in an Update of NN Weights

The vibration-sensitive social characteristic is derived from spiders, one of the most unique species among all kinds of creatures. They make use of a variety of techniques for foraging. However, the model of sensing vibrations is the most effective method of foraging in social spiders. They are susceptible to vibrations, and the vibrations assist them in catching prey. When the observed vibrations of the prey are within a defined frequency, the social spider catches the prey. One of the special features of the social spider is its ability to sense the difference between the vibrations of other social spiders and the vibration produced by prey. The vibration generated by other social spiders helps them to obtain a clear view of the web that effectively reduces the loss of information. The social characteristics can be devised as a cooperative movement of multiple social spiders in reaching the position of the prey. The vibrations from the prey and other social spiders are received and analysed in such a way as to find the optimal position of the prey [25].

The search space of the optimisation problem is initially framed as the hyper-dimensional web of the social spider. All the web positions are considered a feasible solution, and the web acts as a medium of transmission for vibrations. Each social spider occupies a position on the web, and the fitness of the solution depends on the objective of finding the position of the prey. The vibrations contain information about a social spider, which can be used by another social spider on the web. The standard expression of the social spider is generalised as:

(3) $$B_{v}^{q}=\left(L-B_{v}^{q-1}\right)\times rand$$

where $B_{v}^{q}$ is the position of the $v^{th}$ spider at the $q^{th}$ iteration. The above equation can be remodelled as:

(4) $$B_{v}^{q+1}=\left(L-B_{v}^{q}\right)\times rand$$

(5) $$B_{v}^{q+1}=\left(L-0.5B_{v}^{q}-0.5B_{v}^{q}\right)\times rand$$

(6) $$B_{v}^{q+1}=\left(L-0.5B_{v}^{q}-0.5\left[\left(L-B_{v}^{q-1}\right)\times rand\right]\right)\times rand$$

(7) $$B_{v}^{q+1}=rand\left(L-0.5B_{v}^{q}-0.5L\times rand+0.5B_{v}^{q-1}\times rand\right)$$

(8) $$B_{v}^{q+1}=rand\left(L\left[1-0.5rand\right]-0.5B_{v}^{q}+0.5B_{v}^{q-1}\times rand\right)$$

where $B_{v}^{q+1}$ is the position of the social spider in the ${\left(q+1\right)}^{th}$ iteration, L is the lower bound of the search space, and rand is the random floating-point number ranging between 0 and 1. The above equation is the standard equation of the SSO algorithm that is involved in the optimal tuning of the weights of the NN classifier. Algorithm 1 deliberates the pseudocode of the proposed SSO optimisation algorithm.
**Algorithm 1.** Pseudocode of SSO algorithmInitialise the population of social spidersInitialise the target vibrationInitialise the parameters, rand and L
Evaluate the fitness measure for all social spidersFor all social spiders,{Calculate the vibration intensity Select the strongest vibration among all{$n_{\mathrm{int}}>t\arg et_{\mathrm{int}}$Store position of foraging spider v as the best solution}While $q<q_{\max}$
{Update the position of the foraging spider as per Equation (8)}End ForUpdate the parameters, rand and L
Evaluate fitness of all social spidersSort the positions as per fitness measure (accuracy)q=q+1}End ForReturn $B_{v}^{q+1}$


## 4. Results and Discussions

The outcomes of the proposed cancer disease diagnosis module and the comparative study for proving the performance of the proposed NN classifier in disease cancer disease diagnosis is discussed in this section.

### 4.1. Experimental Setup

**Figure 4:**
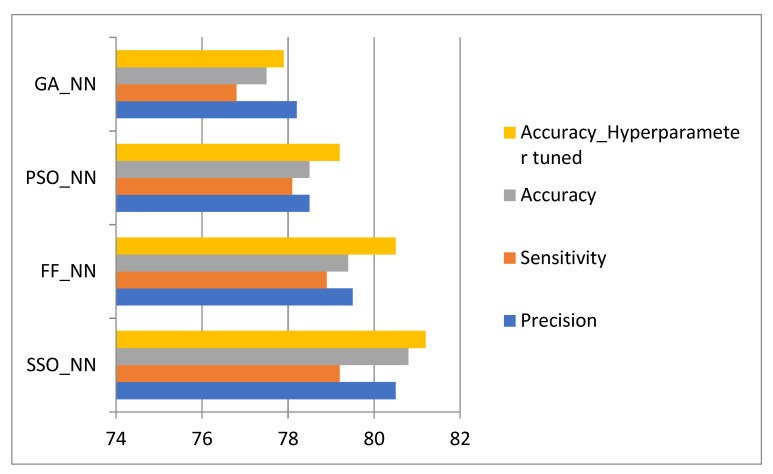
Performance comparison of models.

The experimentation was done in the MATLAB tool installed on a Windows 10 64-bit operating system with 16GB of RAM. The dataset included biopsy images of 58 Hematoxylin and Eosin (H&E)-stained microscopic images of breast tissues (UCSB centre for bio-image informatics)—31 benign and 27 malignant images. The images were augmented to increase the count of samples. Augmentations included standard techniques such as rotation, shearing, zooming, cropping, flipping, and filling, resulting in a total image count of 350 images. In addition to the collected data, the Breast Cancer Histopathological Database (BreakHis) [26] was used to increase the dataset size. The dataset comprised around 9109 microscopic images of breast tumours collected from 82 patients with varying magnifying factors, such as 40, 100, 200, and 400. For validation, however, only 40× magnification images were considered, consisting of 652 Benign and 1370 Malignant images, for a total of 1995 images. The dataset contained four histologically distinct types of benign breast tumours—adenosis (A), fibroadenoma (F), phyllodes tumour (PT), and tubular adenoma (TA)—and four malignant tumours (breast cancer)—carcinoma (DC), lobular carcinoma (LC), mucinous carcinoma (MC) and papillary carcinoma (PC). Hence, the total dataset of 2345 images was split into test data sizes of 25%, and 75% of the data was taken as a training set. After performing the model accuracy, the hyperparameters were tuned using five-fold cross-validation by a gridCV search, which improved the results before the hyperparameter tuning as shown in Figure 4.

### 4.2. Evaluation Metrics

The effectiveness of the proposed cancer diagnosis method was tested using the following measures.

*(a) Accuracy:* The system’s accuracy is evaluated as the rate of the closeness of the obtained quantity to the real quantity. It is expressed mathematically as:

(9) $$Accuracy=\frac{True\;positive+True\;negative}{real\;positive+real\;negative}$$

*(b) Sensitivity:* The sensitivity is the proportion of true positive values to the number of real positive cases. It is expressed mathematically as:

(10) $$Senitivity=\left(\frac{True\;positive_{}}{no\;of\;real\;positive\;cases}\right)$$

*(c) Specificity:* This is characterised as the proportion of true negatives to the count of real negative cases and formulated as:

(11) $$Specificity=\left(\frac{True\;negative_{}}{no\;of\;real\;negative\;cases}\right)$$

*(d) Matthew’s correlation coefficient:* The measure of MCC can be calculated using the expression:

(12) $$MCC=\frac{\left(True\;positive\times TrueN\;negative\right)-\left(False\;\;psoitive\times False\;\;negative\right)}{\sqrt{\begin{array}{l} \left(True\;\;positive+False\;\;positive\right)\times \left(True\;\;positive+False\;\;negative\right)\times \\ \left(True\;\;negative+False\;\;positive\right)\times \left(True\;\;negative+False\;\;negative\right) \end{array}}}$$

### 4.3. Comparative Analysis of Methods Involved in the Diagnosis of Cancer

The methods considered for comparison are the genetic algorithm (GA) [27], particle swarm optimisation (PSO) [28], and firefly algorithm (FF) [29].

The GA-NN, PSO-NN, FF-NN, and the proposed method have shown a considerable increase in accuracy in the specified order. The results do not show much variation in accuracy, since the accuracy of medical images and their thresholds are very important.

**Figure 5:**
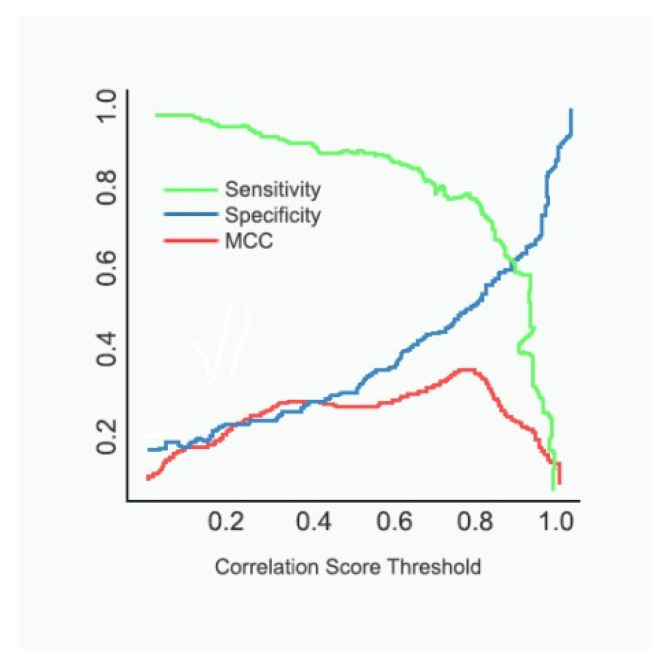
MCC, sensitivity, specificity vs threshold.

**Figure 6:**
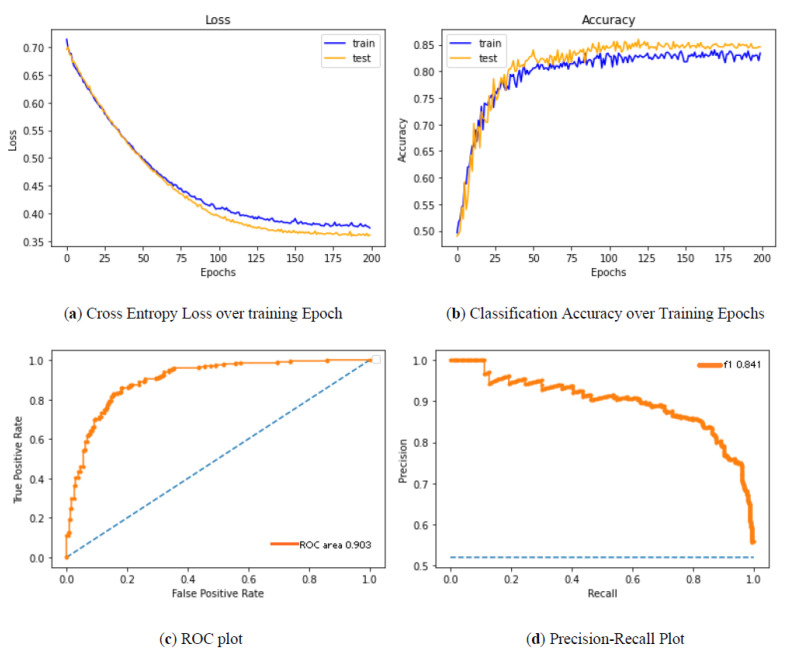
(**a**–**d**) Performance analysis of the proposed method.

In Figure 5, we show the MCC vs. threshold on the same plot as the sensitivity (TP/(TP+FN)) and specificity (TN/(TN+FP)) for the selected data. We see that the peak of the MCC occurs where the specificity is somewhat more significant than the sensitivity. A user might move the classification threshold somewhat lower or higher depending on whether it is more important to retrieve all or practically all true positives, or whether it is somewhat more essential to ensure that the positive results are not contaminated with false positives. It could be observed that the sensitivity drops as the threshold value increases, whereas the specificity increases with an increase in the threshold. The results of Figure 6 descrbes the decrease in loss and increase in classification accuracy as the epoch progresses.

## 5. Conclusions

This paper proposes an artificial intelligence (AI)-based classification model for cancer diagnosis using microscopic biopsy images. The input image is initially subjected to pre-processing to remove artefacts present in the image. The pre-processed image is subjected to watershed segmentation to extract nucleus-based morphological features. Then, the features that include area, perimeter, diameter, and compactness from the nucleus-based morphological features are extracted. The extracted features act as the input to the proposed NN classifier that involves diagnosing the cancer disease. The weights of the NN classifier are optimally tuned using the SSO algorithm to enhance the performance of the proposed model. The proposed strategy’s effectiveness was analysed with performance indices, namely accuracy, sensitivity, specificity, and MCC measures. The measures of accuracy, sensitivity, and MCC were 95.9181%, 94.2515%, and 97.68%, respectively, which shows the effectiveness of the proposed method for effective disease classification. In the future, the NN classifier weights will be optimally tuned by hybrid optimisation algorithms to further increase the accuracy of the proposed model of cancer disease diagnosis.

## Data Availability

The data taken up for analysis includes some self retrieved samples and data from https://web.inf.ufpr.br/vri/databases/breast-cancer-histopathological-database-breakhis/ (accessed on 11 November 2021).
